# Anatomy and computational modeling of networks underlying cognitive-emotional interaction

**DOI:** 10.3389/fnhum.2013.00101

**Published:** 2013-04-02

**Authors:** Yohan J. John, Daniel Bullock, Basilis Zikopoulos, Helen Barbas

**Affiliations:** ^1^Neural Systems Laboratory, Boston UniversityBoston, MA, USA; ^2^Department of Psychology and the Center for Computational Neuroscience and Neural Technology, Boston UniversityBoston, MA, USA

**Keywords:** amygdala, orbitofrontal cortex (OFC), thalamic reticular nucleus, computational neuroscience, neural network, emotions, cognition, neuroanatomy

## Abstract

The classical dichotomy between cognition and emotion equated the first with rationality or logic and the second with irrational behaviors. The idea that cognition and emotion are separable, antagonistic forces competing for dominance of mind has been hard to displace despite abundant evidence to the contrary. For instance, it is now known that a pathological absence of emotion leads to profound impairment of decision making. Behavioral observations of this kind are corroborated at the mechanistic level: neuroanatomical studies reveal that brain areas typically described as underlying either cognitive or emotional processes are linked in ways that imply complex interactions that do not resemble a simple mutual antagonism. Instead, physiological studies and network simulations suggest that top–down signals from prefrontal cortex realize “cognitive control” in part by either suppressing or *promoting* emotional responses controlled by the amygdala, in a way that facilitates adaptation to changing task demands. Behavioral, anatomical, and physiological data suggest that emotion and cognition are equal partners in enabling a continuum or matrix of flexible behaviors that are subserved by multiple brain regions acting in concert. Here we focus on neuroanatomical data that highlight circuitry that structures cognitive-emotional interactions by directly or indirectly linking prefrontal areas with the amygdala. We also present an initial computational circuit model, based on anatomical, physiological, and behavioral data to explicitly frame the learning and performance mechanisms by which cognition and emotion interact to achieve flexible behavior.

## 1. Introduction: integrating emotion and cognition into adaptive perception-action loops

The debate on the nature of cognition and emotion is a modern scientific manifestation of an age-old dichotomy. “Cognition” has come to refer to an assortment of useful behaviors—such as attention, memory, and symbolic reasoning, while “emotion” carries with it the connotation of behavior that is irrational, evolutionarily ancient, and antithetical to efficient rationality. In this paper we outline findings that demonstrate both functional and anatomical overlap between cognitive and emotional processes, and use computational modeling to illustrate how learning processes may make cognitive-emotional interactions adaptive. We focus on a computational neural network model of the amygdalar local circuit, a key hub embedded in a larger system that integrates cognitive and emotional processes.

We begin by describing a plausible functional perspective to frame cognition and emotion as subcomponents of a unified system devoted to categorize bodily and environmental “inputs,” and link the categorized inputs with appropriate behavioral “outputs.” A typical episode of mental life involves three distinctive, but interacting cognitive steps, and each one can vary in complexity: “identifying X, evaluating it as Y, and preparing for behavior that is suitable for X as Y” (cf. Pessoa, 2010). Although the third step, preparation for behavior, is quite diverse, it usually involves at least heightened attention, intentional indifference, or active ignoring. The first and third steps are often labeled with a cognitively-loaded term, e.g., “object categorization” or “stimulus recognition” for step one, and “strategy” or “plan” for step three. The second step, which involves evaluating the present-time significance of X for the agent, is more often labeled with a less cognitively-loaded term such as “affective evaluation,” “emotion,” or “visceral reaction,” depending on the intensity of the positive or negative evaluation. Nevertheless, all three steps can be regarded as cognitive, because all are facets of the agent's “coming to know” (the meaning of the root of “cognition”) and all steps are capable of being corrected, if in error, by further experience. Thus, an object may be briefly misidentified as X until further experience leads to a re-categorization; a stimulus X may be initially construed as an omen of future outcome Y, but re-construed as irrelevant when Y repeatedly fails to follow X; and a plan of action suitable for responding to X as Y in one setting may need to be revised to become effective in another setting.

Though reason and emotion have been viewed as opposed processes in popular culture since ancient times, emotions have been treated as adaptive behavioral phenotypes by scientists since the time of Darwin (1872). Treating emotion as an adaptive phenotype fundamentally subverts any reason-emotion antithesis, because it places emotion as another, if distinctive, enabler of “biological rationality” (Damasio, 1994). Animals have a complex array of cognitive operations to draw upon, and an animal is rational if it knows or can learn how to draw upon those operations to maximize its well-being and minimize threats. In recent years, neuroscientists have shown that the parts of the brain that are recruited during episodes with emotion-arousing stimuli are also de-recruited when no emotion arousing stimuli are present, or when an animal learns that formerly emotion-arousing cues can be safely ignored (e.g., LaBar et al., 1998; Sehlmeyer et al., 2009; Bach et al., 2011; Hartley et al., 2011; van Well et al., 2012). Emotion is indeed a highly adaptive behavioral phenotype.

To better understand cognitive-emotional interactions, we have begun to develop “full-cycle” learning models that explicate how an animal uses its experiences to “come to know” when to engage, disengage, and re-engage its emotional evaluations, to maximize its well-being, and minimize threats, in a highly context-dependent way. Studies of repeated full cycles of acquisition and extinction of Pavlovian associations, as well as studies of repeated learning of experimenter-reversed instrumental (act-outcome) associations, generally show that very little of the associative memory formed during initial acquisition is lost during extinction or reversal phases (e.g., Schoenbaum et al., 2007; Stalnaker et al., 2007). Instead, the neural control system is thought to recruit further pathways that are capable of selectively preventing the expression of prior learning, thus leaving the underlying memory intact.

Otherwise well-regarded formal learning models (e.g., Rescorla and Wagner, 1972) have been incapable of explaining full-cycle learning, because they incorrectly treat extinction as a process that erases most or all of the specific associative memories formed during acquisition (Pearce and Bouton, 2001). For example, neural variants of such models have usually assumed that memories are coded in experience-sensitive synaptic weight values, and that these values greatly increment during acquisition, but severely decrement during extinction training. Although bi-directional synaptic adjustments have been observed during learning protocols at many central synapses (e.g., Diamond et al., 2005; López de Armentia and Sah, 2007; Müller, et al., 2009; Dalton et al., 2012), a model using only the decrementing of learned weights for extinction cannot readily explain data on memory preservation. Notably, reacquisition following even very protracted extinction is much faster than initial acquisition (Napier et al., 1992; Ricker and Bouton, 1996), a phenomenon referred to as “savings” because much of the prior learning is saved from erasure by the extinction process. However, there may be exceptions to this avoidance of erasure. For example, studies in humans suggest that there may be a window of opportunity during which the efficiency of extinction can be enhanced, reducing or preventing such savings (Schiller et al., 2008, 2009).

Our treatment of emotions as part of the rational apparatus of the brain does not preclude also treating emotions as potential sources of irrationality. Emotions as such can lead to maladaptive decisions and behavior if either the learning processes for engaging and disengaging emotions, or the auto-regulatory circuits for controlling the intensity and duration of emotions, are or become dysfunctional. Here the study of full-cycle learning models, suitably rooted in the real circuitry of the brain, should be able to make pivotal contributions. For example, certain learned attractions and fears become obsessive, and extremely resistant to spontaneous reduction. If we understand the full set of processes that enable the normative (i.e., highly flexible and experience-responsive) use of emotional evaluations, then we will also understand which parametric variations of such processes lead to dysfunctions; and we will be able to classify the distinct types of dysfunction. The latter is key for designing minimal-side-effect interventions (whether behavioral, pharmacological, or a mix) that are tailored to the problem. The model introduced here is already illuminating in this regard. Below we present the computational learning model after an overview of forebrain circuits implicated in flexible emotional evaluations, including key structures used in the model. Thus, we constrain and complement the high-level functional approach with an examination of the underlying neuronal circuitry. Using the structural model for connections (Barbas and Rempel-Clower, 1997), we can infer the flow of information relating to sensation, cognition, and emotion along neural pathways. We also describe how frontal cortical regions interact directly and indirectly with the amygdala, the largely subcortical structure most often implicated in emotional processes. Thus, we connect cognition and emotion in two ways—(1) functionally, as equal partners in enabling a continuum or matrix of processes required for adaptive, flexible behavior, and (2) neurally, via diverse cortical and subcortical pathways.

These functional and anatomical perspectives are then integrated via computational modeling. We demonstrate how a neural network model sheds light on the possible mechanisms by which frontal cortical areas influence emotional processing in the amygdala, using classical fear conditioning in the amygdala as an example. Physiological studies from humans and primates are incomplete for the amygdalar circuit, so we also refer to the rodent literature to guide our specification of the model. The amygdalar circuit has rarely been modeled computationally, and therefore we began the computational component of our study here. In recent years this circuit has been delineated in increasing detail, and its complex dynamics are beginning to be understood. Our modeling approach is designed to address some basic questions about emotional learning and behavior. What are some of the implications of the connectivity of the amygdalar local circuit? How does the connectivity allow the system to learn fear associations, and also learn to suppress them when appropriate? Is the amygdalar circuit simply a generator of responses and a repository of emotional memories, or can it participate in information-processing? How might top–down modulatory signals from prefrontal cortex affect the system? In addition to shedding light on these questions, model simulations capture past experimental findings, despite being a schematized approximation of the real amygdalar circuit.

## 2. Roles of the amygdala in emotional processing and learning

Pioneering work on the effects of lesions on the behavior of animals led to the gradual uncovering of emotion-related brain regions (reviewed in (Maren, 2001). This work was stimulated in part by Darwin (1872), who was among the first to place emotion in a biological setting, arguing that emotional states in both humans and animals correspond with neurological phenomena related to movement. The Greek word for emotion (συγκ*í*νηση) also refers to movement. The temporal lobe was the first brain region to be associated with emotional processing (Brown and Schäfer, 1888; Klüver and Bucy, 1937). Removal of the temporal lobe produced marked changes in behavior. Papez (1937) integrated earlier work to propose that an ensemble of linked structures including the hypothalamus, the cingulate gyrus, the hippocampus, and the anterior thalamus form the anatomical basis of emotions (Cannon and Britton, 1925; Cannon, 1927; Bard, 1928). Subsequent work established that the amygdala is also a key element in what came to be known as the Papez–MacLean limbic model (Papez, 1937; Spiegel et al., 1940; Bard and Mountcastle, 1948; MacLean, 1949; Weiskrantz, 1956).

Studies in humans and other animals employing a variety of experimental methods, have provided further evidence on the role of the amygdala in emotion (LeDoux, 1992; Kalin et al., 2004; McGaugh, 2004; Vuilleumier, 2005). The amygdala appears to be necessary for Pavlovian fear conditioning, playing a role in acquisition and expression of fear responses (Maren, 2001), and in the maintenance and retrieval of fear-related memories (e.g., Erlich et al., 2012). But the amygdala is no longer seen as dedicated solely to negative emotions—it also appears to play a role in appetitive conditioning tasks (Everitt et al., 2003), consistent with findings from functional imaging suggesting a role in positive emotions (reviewed in (Fossati, 2012).

The amygdala serves as an important recipient of converging projections from much of the cortical mantle, the hypothalamus, the hippocampus, the brain stem, and the neuromodulatory systems (reviewed in (Sah et al., 2003; Pessoa, 2008). Thus, the connectivity suggests that the amygdala is in a position to contribute to the categorization of the overall state of the organism by integrating information from the body and the external environment. Such categorical representations can then affect sensory, motor, executive, and memory-related processes via the amygdala's diverging outputs (McGaugh, 2002; Sah et al., 2003; Vuilleumier et al., 2004; Hadj-Bouziane et al., 2008). The amygdala can mediate widespread effects via projections to cortical areas (especially prefrontal cortex and the medial temporal lobe), as well as the striatum, nucleus accumbens, thalamus, hypothalamus, and the neurotransmitter systems (Cardinal et al., 2002; Whalen and Phelps, 2009), i.e., the cholinergic, dopaminergic, noradrenergic, and serotonergic structures. Within the forebrain, these projections are strongly implicated in attention, learning, and memory (e.g., Kilgard and Merzenich, 1998; Bao et al., 2001; McGaugh, 2004; Hasselmo, 2006; Parikh and Sarter, 2008; Miasnikov et al., 2009; Ramanathan et al., 2009; Froemke and Martins, 2011; Chau and Galvez, 2012; Medalla and Barbas, 2012).

**Figure 1:**
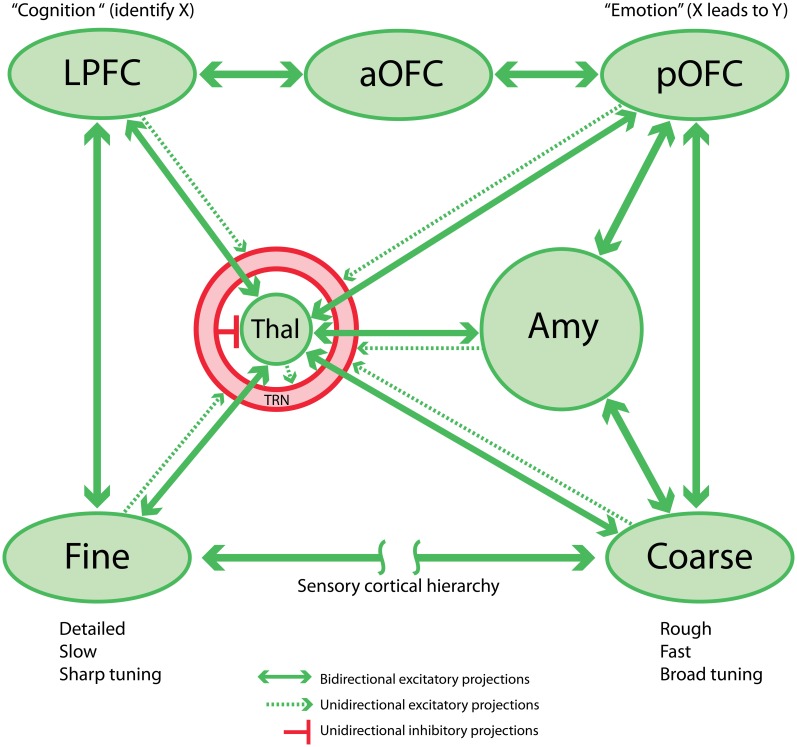
**Schematic circuit linking sensory cortex, prefrontal cortex, thalamus, and amygdala.** This network is the proposed basis for the sensory aspect of the cognition-emotion continuum or matrix that facilitates flexible, adaptive behaviors. Note that the TRN is shown as a shell around the thalamus.

In summary, a coarse-grained survey of amygdala connectivity suggests that it is in a position to influence, and be influenced by, a variety of neural processes necessary for flexible behavior (see (Barbas et al., 2011). The amygdala has a “panoramic view” of internal and external context, and appears to be instrumental in the adaptive control of behavioral states, some of which correspond with emotions (Figure 1). The posterior orbitofrontal cortex (pOFC) has a similarly wide-angled view of body and environment (Barbas, 1995). Perhaps unsurprisingly given this connectional similarity, the pOFC is also implicated in emotional processing, and was incorporated into the Papez–Maclean circuit by Yakovlev (1948) and Nauta (1979). Imaging studies in human patients suffering from post-traumatic stress disorder (PTSD), phobias and social anxiety disorder suggest amygdalar involvement in emotion, particularly negative emotions (e.g., Etkin and Wager, 2007; Nitschke et al., 2009). To demonstrate how the amygdalar circuit is situated within a larger cognition-emotion continuum or matrix, below we review the interactions among prefrontal cortical regions, particularly the pOFC, and the amygdala.

## 3. Neural substrates for cognitive-emotional interactions: pathways through orbitofrontal cortex and the amygdala

The following overview of pathways linking structures associated with cognitive and emotional processes in the mammalian brain has two objectives. First, to outline the essential neural structures used for the model that follows. Second, to demonstrate the need for a model in view of the complexity of the connections. This overview focuses on the intricate connections between the orbitofrontal cortex and the amygdala, regions classically associated with emotion, and lateral prefrontal cortices, which are thought to be key mediators of cognition. The cingulate gyrus and the pOFC were the first prefrontal cortical regions to be associated with emotions (Papez, 1937; Yakovlev, 1948; Nauta, 1979). Both orbitofrontal and anterior cingulate cortices (ACC) are connected with lateral prefrontal cortices. The circuits suggest that these neural structures have a profound influence on each other, inextricably linking emotion and cognition (Barbas, 1995, 2000b; Rolls and Grabenhorst, 2008; Fox et al., 2010; Shackman et al., 2011). This linkage is necessary for normal function and its disruption is at the root of a wide variety of psychiatric disorders.

The circuitry that links pOFC with the amygdala suggests a role in forming emotional associations needed to navigate in a complex and potentially dangerous environment, and in overriding these associations when they are no longer relevant in behavior. Which specific pathways support the flexible formation of emotional associations and their disengagement, as needed? The connectivity alone points to the potential of these circuits to set the system on alert or return it to a quiescent state (Barbas et al., 2003). But the intricacies of these pathways suggest that connectivity alone is not sufficient to infer all their dynamic properties. Computational modeling may assist us in this goal, and also serves as a natural conceptual bridge to link anatomy with physiology. Here we describe the key experimentally determined pathways, providing the framework of a model to address the issue of forming flexible associations.

The posterior strip of the orbitofrontal cortex (pOFC) in macaque monkeys is of special interest for several reasons. The pOFC is by far the most multimodal among prefrontal cortices, and likely the entire cortex (Barbas and Zikopoulos, 2006), and may therefore be the chief sensor of the environment, a cortical counterpart of the older and mostly subcortical amygdala. The pOFC receives information from every sensory system through monosynaptic projections from high-order sensory association cortices including visual, auditory, somatosensory and gustatory cortices, and uniquely from primary olfactory cortices. Further, the pOFC receives robust projections from limbic cortices: the cingulate cortex, the temporal pole, medial (rhinal) temporal cortices and the anterior insula (Barbas, 1993; Carmichael and Price, 1995). We can view the limbic cortices as sensors of the internal, or emotional environment. Based on these connections, the pOFC may be the main cortical sensor of the external and internal environment (Figure 1).

The same sensory association and limbic cortices that project to pOFC also project to the amygdala (Figure 1), which in turn has robust bidirectional connections with the pOFC. This circuitry suggests that pathways from cortices that process environmental signals reach pOFC through a direct route as well as via an indirect route through the amygdala (Porrino et al., 1981; Barbas and De Olmos, 1990; reviewed in, Barbas, 1995, 2000b).

The primate orbitofrontal cortex is connected mainly with the basal amygdalar complex (BLA), composed of the basolateral (BL), basomedial (BM; also known as accessory basal), and the lateral (LA) nuclei (Ghashghaei and Barbas, 2002). Sparser connections are also found with the cortical nuclei of the amygdala. Projection neurons from the basal amygdala innervate most robustly the pOFC as well as the ACC, which forms a crescent at the anterior tip of the corpus callosum. The term ACC here refers to the anterior part of areas 24, 32, and 25 in macaque monkeys. The connections of the pOFC and ACC with the amygdala are bidirectional but not equivalent in each direction (Ghashghaei et al., 2007). The projections from the amygdala to pOFC are stronger than the reciprocal projections, while the opposite is true for the ACC. The ACC sends the most robust return projections to the amygdala.

### 3.1. Flow of information for emotions through sensory cortices, prefrontal cortices, and amygdala

How is information about the external environment evaluated for salience to guide behavior? Information about the entire external environment reaches both the amygdala and pOFC. Sequential pathways from sensory cortices to the amygdala and then to pOFC may supply additional information required to assess the affective meaning of environmental signals. The anatomical reasoning that leads to this proposal begins with the study of the laminar origin of projections from sensory association cortices to the amygdala (Barbas, 2007; Hoistad and Barbas, 2008).

These findings show that sensory association cortices can engage in feedforward signaling to the amygdala, which may in turn categorize the arriving signals based on their affective salience (e.g., Lim et al., 2009; Pourtois et al., 2010a) and convey the results of this categorization to pOFC, where further integration can occur. From the panoramic vantage point of the pOFC, this integrated information is transmitted to the rest of the prefrontal cortex along pathways we examine below. Interestingly, the connections of pOFC and sensory cortices greatly overlap within the basal nuclei in the posterior half of the amygdala, suggesting an efficient passage of salient sensory stimuli from the amygdala to pOFC (Ghashghaei and Barbas, 2002).

Information from the amygdala can thus be followed to pOFC, which is associated with processing the value of stimuli, and from there to lateral prefrontal cortices associated with cognitive processes. This sequence of information processing follows laminar patterns of connections predicted by the structural model for cortico-cortical connections (Barbas and Rempel-Clower, 1997), and tested empirically. In this scheme “feedforward” projections originate from a cortical area that has more layers (or higher neuronal density) than the site of termination. Projection neurons in such a pathway originate in the upper layers and their axons terminate in the middle layers of the receiving cortex. The term “feedback” was originally applied to projections from a later to an earlier processing sensory area (reviewed in (Felleman and Van Essen, 1991). In “feedback” pathways projection neurons are found in the deep layers (5 and 6) and their axons terminate mostly in layer 1. According to the structural model, feedback pathways always originate from areas with fewer layers (or lower neuronal density) and terminate in areas with more layers (or higher neuronal density) than the origin. The terms “feedforward” and “feedback” can be imported to describe connections between non-sensory cortices, via analogy with sensory systems such as the visual system (Barbas, 1986; Barbas and Rempel-Clower, 1997).

The relational rules of the structural model allow prediction of the possible flow of information from pOFC, which receives information about the affective status of the environment, to lateral prefrontal cortices, which are associated with cognitive processes. To begin with, the amygdala innervates all layers of pOFC, including the middle layers (Ghashghaei et al., 2007), which receive feedforward signals. The pOFC projects to lateral prefrontal cortices through sequential steps involving areas with increasingly better defined laminar structure, through anterior orbitofrontal areas and then lateral prefrontal areas, culminating in posterior lateral prefrontal areas 46 and 8, in that order (Barbas and Pandya, 1989). Posterior lateral areas have the best laminar definition within the prefrontal cortex. Functionally they are associated with cognitive processes. The sequential connections follow the rules of the structural model, each stage from pOFC onwards providing sequentially feedback projections to more differentiated (eulaminate) cortices. These pathways suggest that information from the pOFC reaches areas associated with cognitive processes, via successive feedback projections. Physiological data also support this pattern of information flow (e.g., Wallis and Miller, 2003; Bar et al., 2006). Interestingly, feedback projections, which reach layer 1 in all areas, also reach layer 2 and the upper part of layer 3 in most cortices, which collectively make up the upper layers. Layer 2 in several prefrontal cortices is a major target of projections from the amygdala as well (Ghashghaei et al., 2007).

The above linkages suggest an efficient flow of information along sequential feedback pathways from areas with a key role in emotions to areas associated with cognition, decision, and action. The sensory information to the pOFC originates from high-order sensory association areas. The projections from visual and auditory cortices, for example, originate mostly in anterior temporal cortices, which have large receptive fields and likely provide an overview—but not high-accuracy categorizations (Freedman et al., 2003; Freedman and Miller, 2008)—and only modest detail of the external sensory environment (Figure 1, **“Coarse”**). Such a system is suited to quick detection and transmission of coarse-grained or “low-resolution” information, just detailed enough to trigger actions that are imperative to the animal's survival. But what about situations where fine detail about the sensory environment is necessary? Lateral prefrontal cortices are implicated in detail-dependent categorizations (Freedman and Miller, 2008), and these cortices receive “high-resolution” projections, originating from areas representing the external environment, especially visual and auditory association cortices (reviewed in (Barbas, 2000a; Barbas et al., 2002). In contrast to pOFC, lateral prefrontal areas 8 and 46 receive projections from a wide variety of visual cortices, including robust projections from early processing sensory areas adjacent to the primary areas (Barbas and Mesulam, 1981; Barbas, 1988; Schall et al., 1995; Figure 1, “**Fine**”). Early processing visual areas may provide detailed information about the sensory environment. Lateral prefrontal areas also project via two or three steps to orbitofrontal cortices, innervating mostly the middle layers in a feedforward manner. The middle layers in most cortices include the lower part of layer 3, layer 4, and the upper part of layer 5. Projections from pOFC to the amygdala originate overwhelmingly from the upper part of layer 5, which receives feedforward projections from lateral prefrontal cortices.

The laminar pattern of connections thus suggests an efficient way to provide not only a quick overview of the environment to pOFC, but potentially also detailed information through projections from lateral prefrontal cortices. The communication between pOFC and lateral prefrontal cortices is important. The posterior lateral prefrontal cortices are strategically situated in front of the cortical premotor/motor system, poised to guide action using information gathered about the state of the external environment and internal environment through connections with the orbitofrontal cortex and the amygdala (reviewed in (Barbas and Zikopoulos, 2007; Barbas et al., 2011). The pOFC has no direct access to cortical motor control systems.

### 3.2. The pOFC innervates robustly the inhibitory amygdalar intercalated nuclei in macaque monkeys

**Figure 2:**
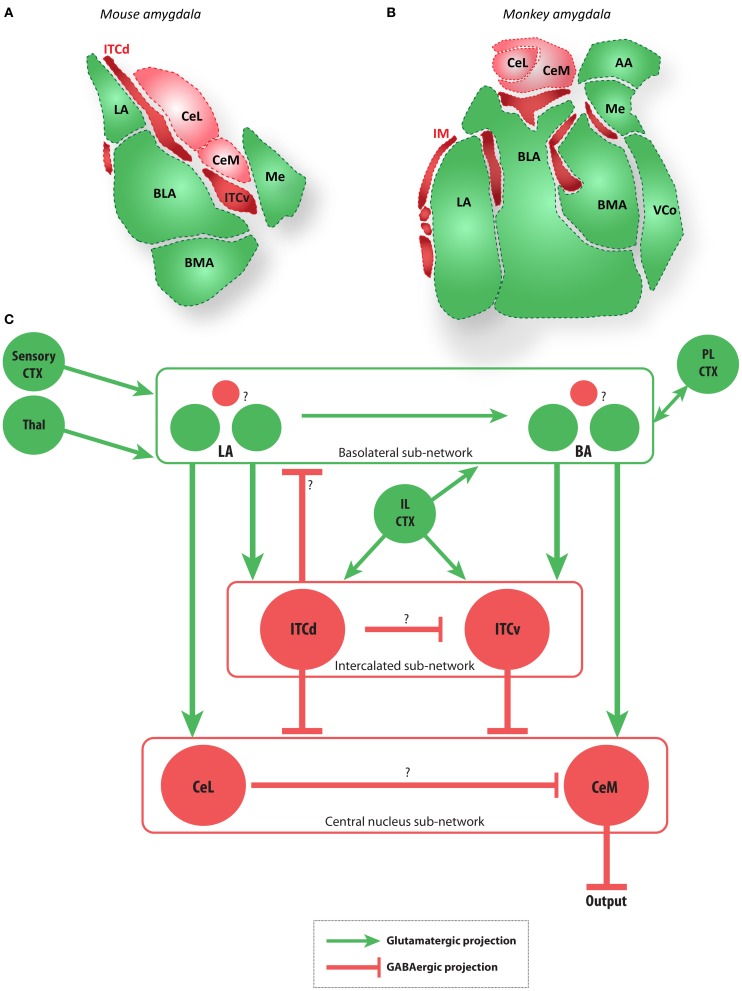
**The amygdala and main extrinsic and intrinsic circuits (A,B)**. Diagrams of coronal sections show key amygdalar nuclei in the mouse **(A)** and macaque monkey **(B)**. Dark red regions are the GABAergic intercalated cells modeled here (ITCd and ITCv in mouse, and IM in macaque), light red regions are the GABAergic CeL and CeM, and green regions are glutamatergic nuclei. **(C)** Schematic depiction of the amygdalar local circuit. The system consists of three components: (1) the cortex-like glutamatergic basolateral sub-network (LA and BA), (2) the striatum-like GABAergic central nucleus sub-network (CeL and CeM), and (3) the GABAergic intercalated sub-network (ITCd and ITCv). The basolateral sub-network receives information about CS and US via projections from sensory cortices (CTX) and thalamus (Thal). In rodents, cortical regions IL and PL project to the intercalated and basolateral sub-networks, respectively. The CeM is a key output node of the network. Question marks indicate local microcircuit details that remain to be fully characterized experimentally. Red circles and lines represent inhibitory cell groups and connections, and green circles and lines represent excitatory cell groups and connections.

The discussion above shows how information from the sensory areas is distilled for valence in the amygdala and passes on to the prefrontal cortex (Ghashghaei and Barbas, 2002; Ghashghaei et al., 2007; Hoistad and Barbas, 2008). We now turn to the reciprocal pathways through which prefrontal cortices may influence the amygdala. In this regard, it is the phylogenetically old (limbic) prefrontal cortices that reciprocate with the most robust return projections to the amygdala (Ghashghaei and Barbas, 2002; Ghashghaei et al., 2007). The pOFC, in particular, has a unique relationship with the amygdala, not shared with any other cortical area: it innervates heavily the intercalated masses (IM) of the amygdala (Ghashghaei and Barbas, 2002), which are composed entirely of inhibitory neurons (Paré and Smith, 1993). In rhesus monkeys the IM nuclei are interposed between the various basal and central nuclei of the amygdala (Figure 2). The significance of the special pOFC pathway is based on the key role of IM within the amygdala, through its projections to the central nucleus (Ce), which is the chief output of the amygdala to autonomic centers (reviewed in (Barbas and Zikopoulos, 2006). The medial part of the central nucleus (CeM), in particular, projects to hypothalamic autonomic structures, as well as to brainstem and spinal autonomic centers and the cholinergic and monoaminergic systems (reviewed in (Sah et al., 2003). The output of the amygdala is in a position to either increase autonomic drive, as seen in emotional arousal, or facilitate return to autonomic homeostasis. The IM nuclei, therefore, appear to be a focal point for the formation of flexible associations in a behavioral setting. Activation of IM may heighten autonomic drive in emotional arousal (Barbas et al., 2003; Pape and Paré, 2010). Alternatively, IM activation may facilitate return to autonomic homeostasis by a mechanism that is not yet clear. In rodents it is the infralimbic (IL) cortex that innervates the inhibitory intercalated nuclei (ITCs). In rats the orbitofrontal cortex does not show the extent of specialization seen in primates. The equivalent region in rats to the primate pOFC is the IL cortex (reviewed in (Vertes, 2006), especially for its projection to the inhibitory intercalated neurons. In rodents, the ITC clusters (ITCs) are thought to have a role in forming emotional associations based on behavioral fear conditioning experiments and physiological studies (Ehrlich et al., 2009; Busti et al., 2011). In macaque monkeys there have been fewer physiological studies on the relevant strip of pOFC and its relationship with the amygdala, but lesion studies suggest that their interactions are similarly important for emotional behavior (e.g., Kalin et al., 2007; Fox et al., 2010). The parallels between the rat and primate circuitry provide the basis for further comparison (Figure 2). The behavioral and physiological findings from rodents and wealth of anatomical data in primates can be linked via a computational model based on their circuit commonalities. Ongoing research may also point out differences between the circuits, and what these differences imply about generalizing the conclusions of emotional learning studies in rodents to primates and humans.

### 3.3. The role of the anterior cingulate

In the above discussion of cortico-cortical connections we have not considered in any detail the role of the ACC in the process of linking areas associated with emotional and cognitive processes. Like the pOFC, the ACC is part of the prefrontal limbic system (Vogt et al., 2005), and has strong connections with the amygdala as well (Ghashghaei et al., 2007). However, it differs from the pOFC in several ways. The ACC does not have the exquisite focal projection to the inhibitory IM nuclei in primates, and it lacks multimodal connections (Barbas et al., 1999) that are characteristic of the pOFC. In fact, with the exception of connections with auditory association cortices, the rest of the unimodal sensory association cortices do not have significant projections to the ACC. But the ACC has its own specializations (e.g., Buckley et al., 2009; Pourtois et al., 2010b). Among prefrontal cortices it has the strongest connections with the rest of the prefrontal cortex, and is well suited to allocate attentional resources, as is widely reported (see Medalla and Barbas, 2009, 2010; reviewed in, Burgess et al., 2000; Paus, 2001; Rushworth et al., 2007). In addition, the ACC receives strong monosynaptic projections from the hippocampus, and has bidirectional connections with medial temporal cortices (Bunce and Barbas, 2011), in pathways that are thought to convey contextual information (reviewed in (Barbas et al., 2013). The ACC has robust connections with the pOFC, perhaps providing the contextual information necessary to interpret signals in the environment and contribute to emotional arousal. Interestingly, the ACC is the primary effector to brainstem autonomic structures through projections to hypothalamic and spinal autonomic centers (Ongur et al., 1998; Rempel-Clower and Barbas, 1998; Barbas et al., 2003). These features suggest that pOFC is the primary cortical sensor of emotional information, whereas the ACC is the primary effector of emotional expression, linking motor control, cognition and drive (Barbas, 2000a,b; Paus, 2001; Shackman et al., 2011).

## 4. Emotional learning and expression via the amygdala

Below we examine a local circuit in the amygdala implicated in the learning and execution of a widely studied emotional behavior: acquisition and extinction of the fear-potentiated freezing response via Pavlovian conditioning (Pavlov, 1927; Maren, 2001), in which an initially neutral sensory cue (the conditioned stimulus, CS) such as an auditory stimulus is regularly followed by an emotion-evoking stimulus (the unconditioned stimulus, US). These pairings are separated by a much larger inter-trial interval. The functioning of this circuit can be viewed as a form of emotional categorization or salience-assignment. In rats this circuit receives top–down projections from medial prefrontal cortex (IL), which modulate the behavior of the inhibitory ITCs. It is important to note that much of the behavioral and physiological data on fear conditioning come from rodent studies. The degree to which the rodent circuit resembles the primate circuit is presently unclear, but many major connections appear to be similar across species. Diagrams of coronal brain sections in Figure 2 show the amygdala local circuit in the mouse (Figure 2A) and the rhesus macaque (Figure 2B), depicted in schematized form in Figure 2C. After reviewing the transmission of signals through the amygdalar circuit, we demonstrate how computational modeling of this circuit can illuminate the possible mechanisms for top–down control of emotion. This modeling effort suggests possible functional roles for the ITCs that have not yet been explored experimentally.

The following general principles of amygdalar organization have been widely observed in rodents (e.g., Sah et al., 2003; Ehrlich et al., 2009; Pape and Paré, 2010): (1) The BLA nuclei consist of a majority of glutamatergic projection neurons and a minority of local GABAergic interneurons, as in the cortex; (2) the medial structures (Ce) are striatum-like, with the vast majority of neurons being GABAergic, with spiny-type morphology; (3) the internuclear projections generally follow a dorso–ventral and latero–medial direction; (4) the ITCs add an additional layer of complexity as recipients of projections from medial prefrontal areas, and specifically the IL cortex in rats. In rhesus monkeys IM neurons (the primate equivalent of ITCs; see Figure 2) receive projections from pOFC (Ghashghaei and Barbas, 2002). While the layout of the amygdalar circuit elements in primates is broadly similar to that of rodents (Figure 2), the relationship among IM neurons is not yet clear in primates.

Several recent studies of fear conditioning in rats have suggested a flow of information within the amygdala as shown in Figure 2C (reviewed in (Debiac and LeDoux, 2009). Thus, LA is seen as the input station, receiving sensory signals from thalamus and cortex, and the central nucleus (primarily CeM) is seen as the output station, with BA and the ITC clusters serving as intermediate processing stages. Below we examine in more detail the ITCs, which may serve as important loci for cognitive control of emotions.

### 4.1. The role of intercalated neurons and their cortical inputs

The rodent ITCs have emerged as key elements in emotional learning and expression (Ehrlich et al., 2009; Pape and Paré, 2010; Li et al., 2011; Manko et al., 2011; Palomares-Castillo et al., 2012). At least three anatomically distinct groups of ITCs have been identified in rodents. Two of these groups (Figure 2) appear particularly important for fear conditioning and extinction: (1) the dorsal group (ITCd), also called the medial paracapsular group; and (2) the ventral group (ITCv), also called the main intercalated nucleus. For example, Busti et al. (2011) showed that during fear conditioning in mice, the selective activation of ITCd by LA following repeated CS-US pairings triggers feedforward inhibition in ITCv, which disinhibits CeM output neurons and releases a fear response (freezing). Conversely, extinction training, in which the CS is repeatedly presented without a following US, leads to CS activation of ITCv, and suppression of fear responses.

The firing properties of ITCs also suggest possible functional roles exemplified by groups of neurons that fire at much higher rates than commonly observed in neighboring amygdalar sites in unanesthetized cats (Collins and Paré, 1999). Their high spontaneous firing rates suggest that the ITC clusters provide tonic inhibition to their targets. The firing probabilities of ITC neurons are modulated by ecologically salient stimuli, such as cat growling, dog barking, and birdsong. These findings suggest that emotionally or environmentally salient stimuli can alter the firing rates of some ITCs.

Anatomical and physiological studies implicate prefrontal projections in modulation of the inhibitory effects of ITCs. The pOFC in primates (Ghashghaei and Barbas, 2002), and IL in rodents (Berretta et al., 2005; Pinto and Sesack, 2008; Sierra-Mercado et al., 2010; Sotres-Bayon and Quirk, 2010; Pinard et al., 2012) send robust excitatory projections to the inhibitory ITCs. Some evidence suggests that prelimbic cortex (PL) in rats and cats may also project to ITCs. There are also well-established projections from PL (or primate ACC) to BLA and Ce. (reviewed in (Vertes, 2004). Consistent with the inhibitory role of ITCs on amygdalar output, it has been observed that stimulation of medial prefrontal areas in cat and rat decreases the responsiveness of neurons in Ce to inputs from BLA (Quirk et al., 2003).

The projections from cortex to the ITCs also appear to have behaviorally relevant effects on learned engagement and disengagement of fear. For example, Sierra-Mercado et al. (2010) found that in rats, inactivation of IL neurons with muscimol impaired acquisition and retention of fear extinction, but left fear expression unchanged. Muscimol inactivation of PL had the opposite effect: it impaired fear expression but did not disrupt extinction. The IL (analogous to primate pOFC) was more important for learning to engage and disengage fear, whereas PL (analogous to primate ACC) was more important for expressing fear.

In summary, data suggest that ITC neurons play an important role in acquisition and extinction of fear responses. Further, this role appears to be subject to top–down modulation or control from IL cortex in rodents and pOFC in primates. These projections can thus serve as conduits for cognitive modulation of fear expression and suppression.

### 4.2. “teaching signals” within the amygdala

In order to model flexible learning in the local circuit outlined above it is necessary to have plausible neural “teaching signals” that can modify network connections in response to aversive events. Teaching signals, as defined in theories of reinforcement learning (reviewed in (Sutton and Barto, 1998), are signals that co-occur with salient events or prediction errors, and therefore facilitate learning from experience. Neural signals that co-occur with appetitive or aversive events and also facilitate synaptic plasticity, such as phasic changes in firing rate or neurotransmitter release, are typically employed as teaching signals in neural models of reinforcement learning. The amygdala receives convergent pathways that carry information about the CS and about the US to the same zones, wherein associative learning processes assess whether a particular CS is predictive of a particular US. If so, synapses transmitting CS information gain control of emotional responses that are typically evoked by the US. In short, information about the US that arrives in the amygdala constitutes a specific teaching signal for intra-amygdala learning. In the case of learned fear, for example, important US pathways include ascending somatosensory-nociceptive pathways to amygdala (Bourgeais et al., 2001; Lanuza et al., 2004; Johansen et al., 2010; McNally et al., 2011). The co-occurrence of such signals with CS signals can trigger associative learning in the amygdala, which can enable CSs to elicit anticipatory freezing in order to avoid pain. Studies in humans have also demonstrated expectation-related activity in the amygdala (e.g., Sarinopoulos et al., 2006; Pourtois et al., 2010b).

## 5. Computational modeling of the amygdala circuit

We employ computational modeling techniques to understand how the amygdalar circuitry reviewed above can serve as the mechanistic basis for some emotional processes, and how top–down modulatory signals from cortex can influence these processes. In this section we show that a computational model of the amygdalar local circuit described above can exhibit flexible acquisition and suppression of stimulus-triggered emotion-related responses, using classical fear conditioning as a test case. Learning in the model can be interpreted as the categorization or labeling of stimuli based on their affective consequences. Stimuli that have been thus categorized can then drive fear-related behavior such as the freezing response in rodents. Cortical modulation in the model adds some flexibility, so fear-related responding is not an inevitable consequence of presenting categorized stimuli. Our modeling approach provides a simplified coarse-grained perspective on the amygdala local circuit: we implement rate-coding rather than spiking in order to investigate properties of the network that arise from connectivity as opposed to the physiological parameters of particular neuronal types.

The circuit diagrams proposed in prior studies can be combined into a single schematic diagram (Figure 2C) that captures the general flow of information common to many of the anatomical and physiological studies. At least three sub-networks can be distinguished in many of the relevant rodent studies: (1) the BLA sub-network, an input stage consisting of excitatory projection neurons and inhibitory interneurons in LA, BL, and BM; (2) the ITC sub-network, an intermediary stage consisting of at least two sub-populations of inhibitory cells; and (3) the central sub-network, an output stage consisting of inhibitory projection neurons and interneurons in CeL and CeM. Fear-related responses appear to be expressed via excitation of CeM either directly, or via disinhibition. The key external sources of inhibition to the CeM are neurons in the ITCs and in CeL. As reviewed above, extinction and suppression of learned fear responses seem to involve enhancing inhibition from these sources.

### 5.1. Model description

**Figure 3:**
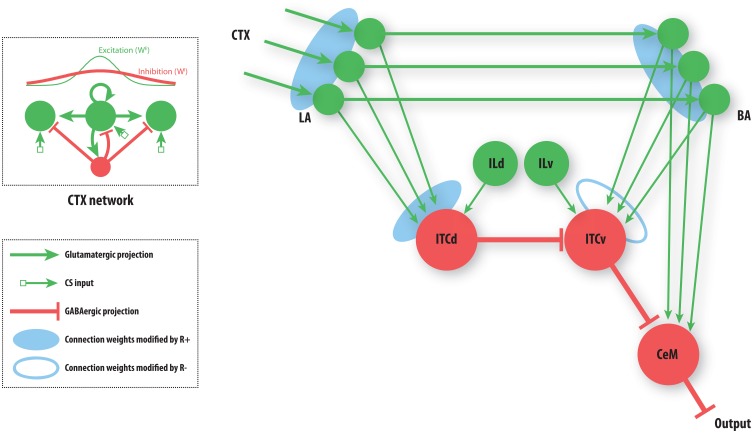
**A simplified amygdala circuit for emotional responding.** An array of stimulus-related excitatory outputs from a cortical network (CTX) projects in a topographic manner to the lateral nucleus of the amygdala (LA). The cortical network (top inset) is constructed as a distance-dependent on-center off-surround network. Amygdalar input station LA sends excitatory projections topographically to the basal nucleus of the amygdala (BA). Thus, for every cortical cell there is a corresponding cell in LA and in BA, and a corresponding weight from LA onto the inhibitory dorsal intercalated cell (ITCd) and from BA onto the inhibitory ventral intercalated cell (ITCv). The whole array of excitatory LA cells converges onto one ITCd cell. Similarly the array of excitatory BA cells converges onto one ITCv cell, and one cell of the central nucleus of the amygdala (CeM). The ITCd cell inhibits the ITCv cell. The ITCv cell inhibits the CeM cell. The ITCd and ITCv cells each receive projections from infralimbic cortex (IL). Green arrows represent excitatory glutamatergic projections. Red flat arrows represent inhibitory GABAergic projections. Blue ovals represent modifiable synaptic weights. The filled blue ovals represent weights that are potentiated by the arrival of the US (*R*^+^). The empty blue oval represents weights that are potentiated by the arrival of a US prediction-error signal (*R*^−^).

We simulate a neural network based on the simplified amygdala connectivity depicted in Figure 3. Rate-coded cell activities representing incoming stimuli such as auditory or visual cues project topographically to LA from the cortex (CTX). We leave out projections from thalamus to LA, but the model would work in similar fashion if equivalent sensory information is also conveyed through the thalamic projections. Cells in LA then project topographically to a similar array in BA. Thus, for every stimulus encoded in CTX there is a corresponding cell in LA and in BA. Cells in LA converge onto a single ITCd cell. Similarly, the array of cells in BA converges onto a single ITCv cell. The ITCd cell inhibits ITCv and CeL. The ITCv cell inhibits CeM, the main output station of the amygdala. This circuit is based on evidence from rodent studies reviewed above.

Each cue (CS) can come to be associated with a negative consequence such as footshock, via classical conditioning. A signal (*R*^+^) that corresponds to the foot shock (an unconditioned stimulus; US) arrives at three network locations, as shown (Figure 3). A signal (*R*^−^) corresponding to the non-occurrence of an expected US arrives only at the ITCv. These two signals gate Hebbian synaptic change, and therefore serve as teaching signals. We will now briefly describe the model's performance, before demonstrating the simulation results.

The learning process causes the potentiation of synapses on LA cells whenever the corresponding CS co-occurs with the US (Erlich et al., 2012). BA cells in turn are potentiated whenever LA activities co-occur with the US. Synapses onto the ITCd cell are potentiated whenever LA activity overlaps with the US. Similarly, synapses onto the ITCv cell are potentiated whenever BA activity overlaps with the absence of an expected US. Depression of synaptic weights onto the ITC cells occurs whenever the conditions for their potentiation are not met. Weights onto the ITCd cell decrease when the CS-US pairing is extinguished, whereas weights onto the ITCv cell follow the opposite pattern: when CS-US pairing is extinguished, the weights of synapses from BA onto ITCv increase, allowing ITCv to suppress previously learned fear responding. Over time, the activities of LA and BA cells co-occur with those CSs that have been paired with the US. Learning is modeled phenomenologically—this captures empirically established rules regarding the experience-dependence of long-term potentiation (LTP) and long-term depression (LTD) at selected amygdalar synapses. (See Methods section for a brief description of our phenomenological modeling approach).

Synaptic depression is assumed to be much faster in the ITC clusters than in LA and BA. Direct evidence for this assumption is not yet available, but physiological findings on ITCs are consistent with it (Pape and Paré, 2010; Busti et al., 2011; Manko et al., 2011). Weights onto cells in LA and BA, once potentiated, are assumed to decay only negligibly over the time scales simulated. The difference in decay rate allows for flexibility in the face of changing contingencies without erasure of previously learned CS-US associations. In the model, weights of synapses onto ITCd and ITCv cells change rapidly, allowing the system to switch from a response mode to a response-suppression mode, without necessitating unlearning at the level of LA or BA. Thus, the synapses onto LA and BA allow for post-extinction savings, whereas synapses onto the ITCs allow for sensitivity to changes in contingency.

**Figure 4:**
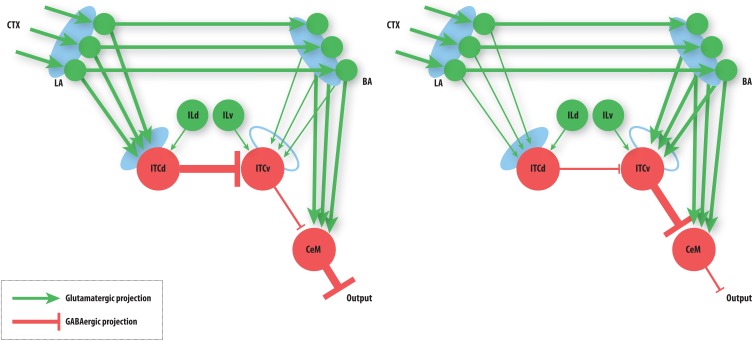
**Summary of basic model behavior.** Line thickness denotes strength of activity. **Left:** The state of the network after fear acquisition. ITCd inhibits the ITCv, thereby disinhibiting CeM, allowing BA to excite it. CeM excitation leads to fear responding. **Right:** The state of the network after extinction. ITCv inhibits CeM, so CeM becomes insensitive to BA excitation. Note that the learning at LA and BA synapses is not lost. CeM inhibition suppresses fear responding. See caption of Figure 3 for key to symbols and a description of the circuit.

The basic performance of the model is as follows (Figure 4): after fear acquisition, weights on LA, BA, and ITCd are potentiated, leading to inhibition of ITCv, and excitation of CeM, the output cell of the network that triggers the fear response. After fear extinction, weights on LA and BA are almost unchanged, but the weights on ITCd have decreased, and weights on ITCv have increased, causing CeM to be inhibited and the fear response to be suppressed. Simulation results also show some degree of redundancy in the ITCd and ITCv synapses. In some situations this apparent redundancy may be unmasked, so the two areas can serve distinct functions. Simulations also reveal a possible information-processing role for the ITCs.

Cortical modulation from IL onto ITCd and ITCv can be used to bias the circuit's behavior toward or away from extinction. IL can be used to enhance the activity of ITCd, thereby increasing inhibition of ITCv and leading to greater disinihibition of CeM. Alternatively, IL can be used to enhance the activity of ITCv, increasing inhibition onto CeM. The IL (pOFC in primates) is thus well placed to bias the information-processing role of the ITCs.

The cortical network (CTX) is structured as a distance-dependent on-center- off-surround shunting network. Networks of this type offer a simple, neurally plausible means of implementing contrast-enhancement, as well as a host of other processes (Grossberg, 1973). The strength of the off-surround inhibition can be varied to determine how sharply the cell activities represent a set of incoming stimuli. In other words, controlling inhibition modulates the tuning curve of each cell. Strong inhibition allows for sharp contrast, whereas weak inhibition leads to spreading activity and lower contrast. This can serve as a simple model of top–down attention. High attention corresponds to sharp tuning or high contrast, whereas low attention can lead to broader tuning or lower contrast. Low contrast can be used to make “fuzzy” representations that can be used as a basis for generalization of stimuli. In the case of fear conditioning, the amygdala circuit can be interpreted as categorizing stimuli as either predictive or non-predictive of an aversive US. High contrast in the CTX will allow the system to accurately respond only to the CSs paired with the US. But the system will not generalize to CSs that have not been presented. For instance, if a sound of a particular frequency is paired with footshock, then a range of similar frequencies will also elicit a fear response. In situations of generalization, the range that is determined to be similar is widened, so more frequencies come to elicit the fear response. Thus, modulating inhibition in the CTX provides a way to investigate the effects of attention or stimulus tuning on fear learning. Some studies of generalization during fear conditioning implicate hippocampal dysfunction (reviewed in (Kheirbek et al., 2012). It may be that an analogous mechanism to the cortical one posited here may be applied to hippocampus-dependent changes in generalization.

### 5.2. Simulation results

The circuit in Figure 3 has many degrees of freedom. Here we focus on the possible roles of the ITC masses ITCd and ITCv in emotional learning. The only weights that are subject to synaptic change are the weights from the cortex to LA, from LA to BA, from LA to ITCd, and from BA to ITCv. All other weights are held constant.

In these results, we ignore the projection from CeL to CeM in order to focus on the inhibitory action of ITCv on CeM. As the network diagram suggests (Figure 2), there is redundancy in the inhibitory pathways to CeM. Simulations (not shown) confirm the idea that CeL and ITCv have very similar roles in the simplified circuit shown in Figure 3. For simplicity we omit the activity of CeL in the results that follow. A more detailed model incorporating additional connections will be necessary to investigate asymmetrical roles for each source of inhibition to CeM.

#### 5.2.1. Fear-related learning

**Figure 5:**
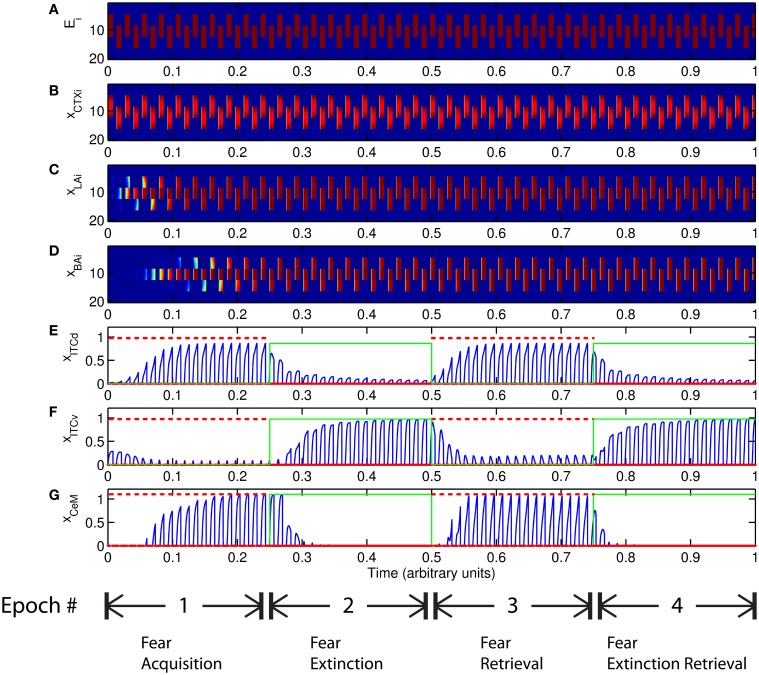
**Time course of model cell activities during “normal” mode.** The system exhibits fear acquisition, extinction, fear retrieval, and extinction retrieval. Plots show the time evolution of model cell activities during four consecutive epochs. Each plot **(A–D)** shows the temporal evolution of an array of 20 cells, with the y-axis representing the cell index, and the color representing strength of activation (Blue is low, red is high). At any given time there is either a CS1-US pairing, a CS2-US pairing, a presentation of CS1 alone, a presentation of CS2 alone, or an intertrial interval. Because the features of CS1 and CS2 overlap, the indices of activation bars for corresponding representations in **(A–D)** also overlap. Each plot **(E–G)** shows the temporal evolution of a single cell, with the y-axis representing strength of activation of that cell. In the first and third epochs, the CS signals, shown in **(A)**, co-occur with *R*^+^, the US. In the second and fourth epochs, the CS signals are presented without *R*^+^. The red dotted lines in **(E–G)** indicate presence of *R*^+^. The green lines indicate extinction epochs in which *R*^+^ = 0. (Parameters: strength of inhibition *f*_*I*_ = 3.0; input from IL to ITCd *E*_ILd_ = 0; input from IL to ITCv *E*_ILv_ = 2).

**Figure 6:**
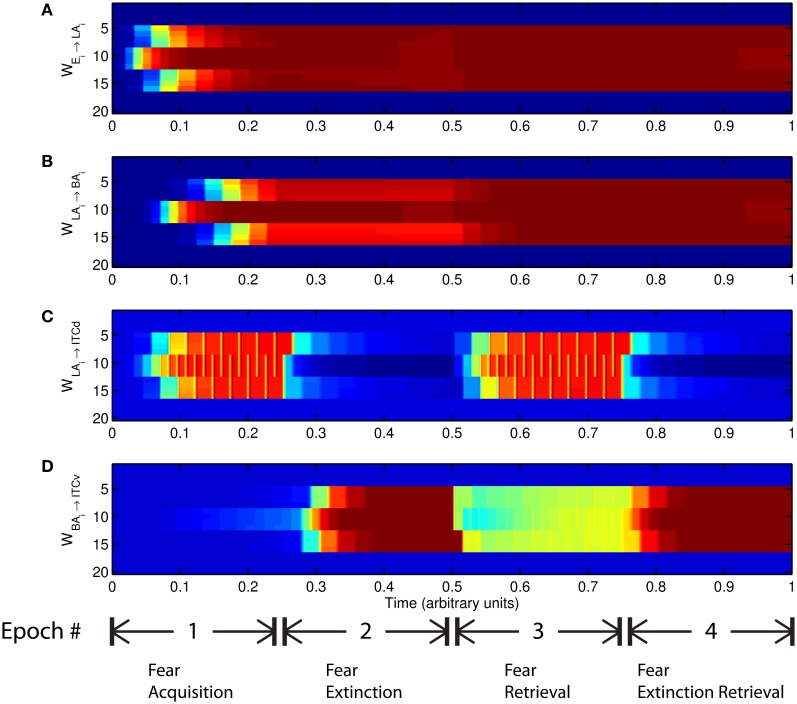
**Time evolution of model synaptic weights during “normal” mode.** The system exhibits fear acquisition, extinction, fear retrieval, and extinction retrieval. Plots show the time evolution of model connection weights during four consecutive epochs. Each plot **(A–D)** shows the temporal evolution of an array of 20 connection weights, with the y-axis representing the index of the corresponding cell, and the color representing connection strength (Blue is low, red is high). In the first and third epochs, the CS signals co-occur with *R*^+^, the US. In the second and fourth epochs, the CS signals are presented without *R*^+^. (Parameters: strength of inhibition *f*_*I*_ = 3.0; input from IL to ITCd *E*_ILd_ = 0; input from IL to ITCv *E*_ILv_ = 2).

The basic behavior of the model is illustrated schematically in Figure 4. The model is taken through four consecutive learning epochs: (1) fear acquisition, (2) fear extinction, (3) fear retrieval (the post-extinction re-engagement of fear responding), and (4) extinction retrieval (the re-engagement of fear extinction). The CTX network receives two CSs with overlapping representations, i.e., the two CSs activate a common subset of cells in the CTX array. For instance, two auditory signals with overlapping frequencies, represented in a tonotopic manner, can be used as the CSs. The two CSs are presented in alternation, and while on, each co-occurs with the US (*R*^+^) during epochs 1 and 3. The prediction-error signal (*R*^−^) takes non-zero values in epochs 2 and 4. The time course of model cell activities is shown in Figure 5. The red plots in E–G indicate *R*^+^ (the US), and the green lines in E–G indicate absence of *R*^+^, or extinction trials. When the CS is shut off (*E*_*i*_ = 0), a trial ends, and the activities are reset to zero. Only the connection weights persist through the intertrial intervals. The corresponding time course of changes to connection weights is shown in Figure 6.

The key development during acquisition is the learning of the CS-US association by the weights onto LA and BA. Once these associations have been formed, they are not significantly weakened during extinction epochs, as is apparent in Figures 6A and 6B, though the modeled synapses can undergo both LTP and LTD at a slow rate.

#### 5.2.2. Using frontal cortex to bias the system

**Figure 7:**
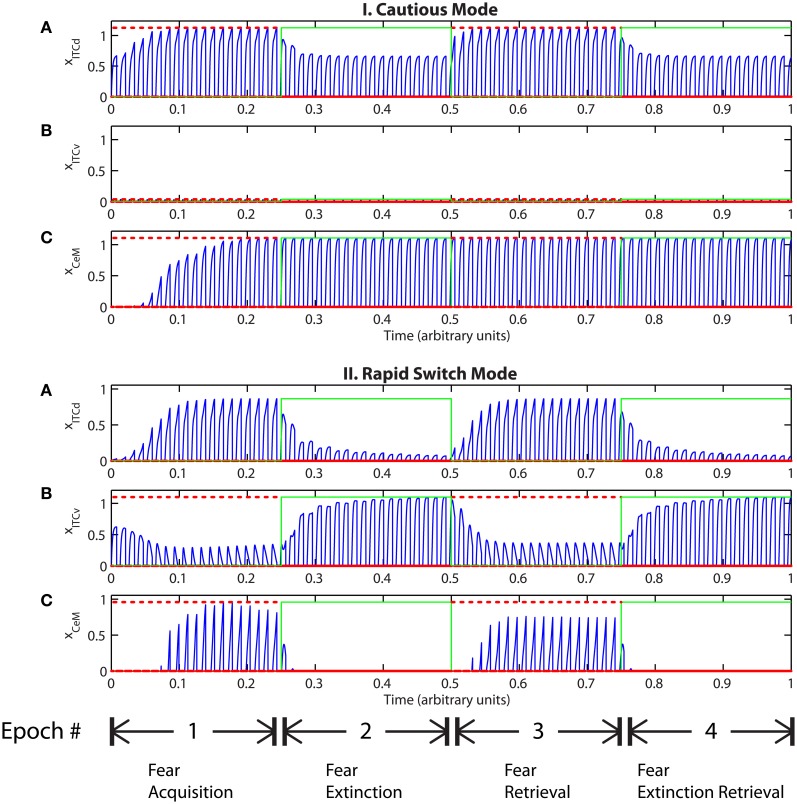
**(I)** Time evolution of model cell activities during “cautious” mode (when ITCd activation is much higher than ITCv activation): the system is biased to prevent extinction. (Parameters: strength of inhibition *f*_*I*_ = 3.0; input from IL to ITCd *E*_ILd_ = 5; input from IL to ITCv *E*_ILv_ = 2). **(II)** Time evolution of model cell activities during “rapid switch” mode (when ITCv activation is much higher than ITCd activation): the system is biased to enhance extinction. (Parameters: strength of inhibition *f*_*I*_ = 3.0; input from IL to ITCd *E*_ILd_ = 0; input from IL to ITCv *E*_ILv_ = 5). Each plot shows the temporal evolution of a single cell over four consecutive epochs, with the y-axis representing strength of activation. In the first and third epochs, the CS signals, co-occur with *R*^+^, the US. In the second and fourth epochs, the CS signals are presented without *R*^+^. The red dotted lines indicate presence of *R*^+^. The green lines indicate extinction epochs in which *R*^+^ = 0.

Tonic excitation from cortex (ILd and ILv) to ITCd and ITCv can be used to bias the output of the circuit, i.e., the activity of CeM. For example, if the net excitatory input to ITCd is above a certain threshold, the system is no longer able to extinguish previously learned fear responding (Figure 7-I). The system has effectively been placed in a “cautious” mode, so during an extinction epoch the system does not suppress the fear-related responses to associations that were formed during the preceding acquisition epoch. Similar results are obtained when the input to ITCv sinks below a threshold. These effects occur due to the inhibitory effect of ITCd on ITCv. If ITCv cannot be driven by cells in BA, it cannot suppress previously learned fear responding. In other words, in configurations of this type, the system is insensitive to weakening of the link between CSs and the aversive US. In a “cautious” mode, the system does not let down its guard, and continues to generate fear-related responses to CSs long after they cease to co-occur with the US.

Conversely, if excitatory signal *E*_ILv_ from ILv to ITCv is high and signal *E*_ILd_ from ILd to ITCd is low, the system rapidly switches from fear responding to no response after fewer extinction trials (Figure 7-II). The system has effectively been placed in a “rapid switch” mode, so during an extinction epoch it can more quickly learn to suppress fear-related responses. These simulations agree with experimental results showing the importance of the ITCs in fear learning and expression. The cortical control of the ITCs may be an important route for top–down cognitive control of emotional learning and behavior, adding additional flexibility to responses that are often considered automatic. Further, because the inputs from ILd and ILv can take continuous values, the system's rate of extinction of fear-related responses—or, conversely, its degree of “caution”—can be smoothly varied between the two extreme states.

#### 5.2.3. Redundant learning

**Figure 8:**
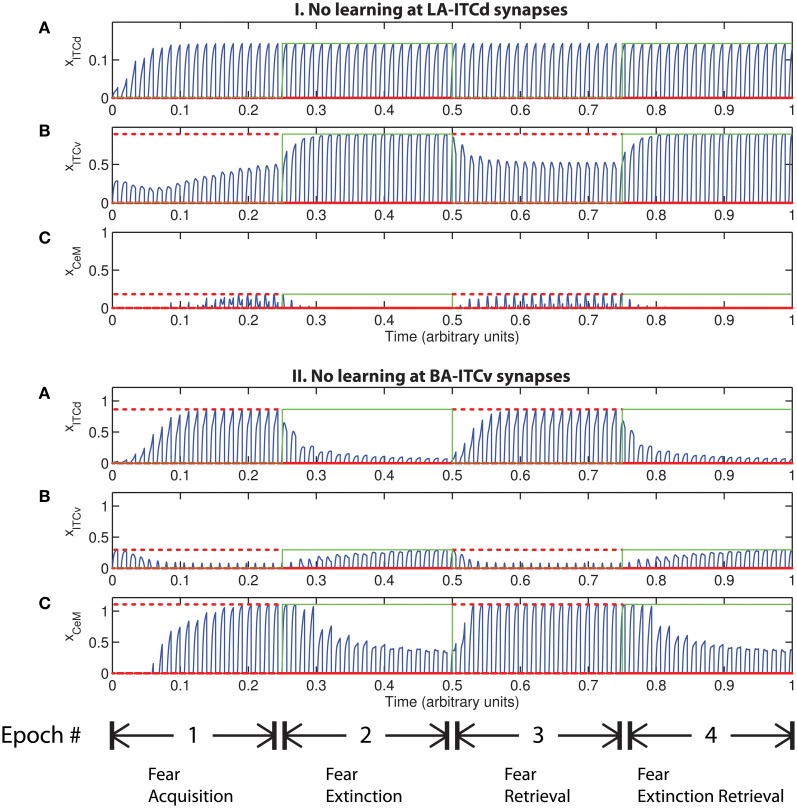
**Time evolution of model cell activities. (I)** No learning occurs at the LA-ITCd synapses. **(II)** No learning occurs at the BA-ITCv synapses. Each plot shows the temporal evolution of a single cell over four consecutive epochs, with the y-axis representing strength of activation. In the first and third epochs, the CS signals, shown in (A), co-occur with *R*^+^, the US. In the second and fourth epochs, one of the CS signals is presented without *R*^+^. The red dotted lines indicate presence of *R*^+^. The green lines indicate extinction epochs in which *R*^+^ = 0. (Parameters: strength of inhibition *f*_*I*_ = 3.0; input from IL to ITCd *E*_ILd_ = 0; input from IL to ITCv *E*_ILv_ = 2).

An important question that arises when modeling a given circuit is whether all the network's dynamic processes are required to produce the same qualitative output, or if a subset will suffice. In some circumstances the weights onto ITCv appear to encode redundant information. The apparent redundancy can be demonstrated by turning off learning in the ITCv and ITCd weights one by one. In Figure 8-I there is no learning in the LA-ITCd weights, so the amplitude of the CeM response is weakened, resulting in failure to acquire an appreciable fear response to CS presentation. In Figure 8-II there is no learning in the BA-ITCv weights, and the CeM response is normal during acquisition trials. During extinction epochs there is some reduction in the CeM reponse, but it is partial. Thus, the learning at BA-ITCv synapses may appear redundant, as a reduction in CeM response amplitude can be achieved without it.

**Figure 9:**
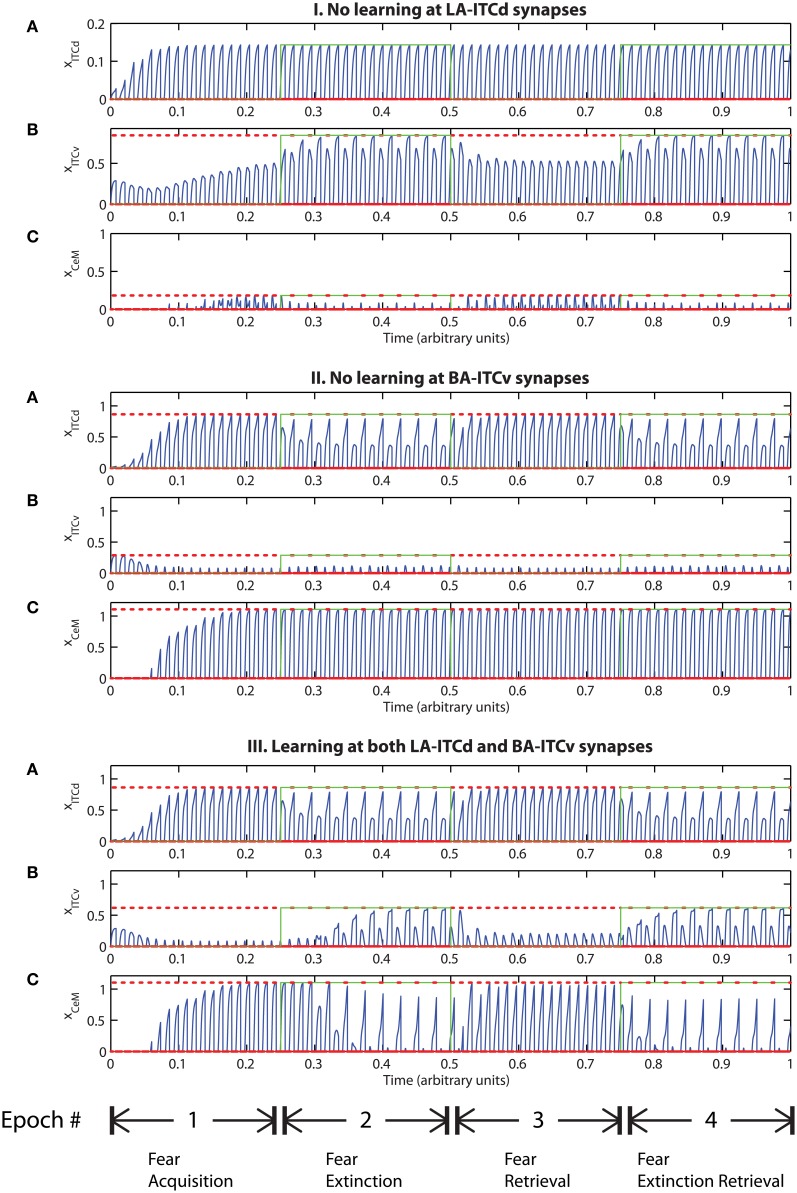
**Time evolution of model cell activities during “confusing” outcomes.** Only one of the two overlapping CS signals is extinguished. **(I)** No learning occurs at the LA-ITCd synapses. **(II)** No learning occurs at the BA-ITCv synapses. **(III)** Learning occurs in both LA-ITCd and BA-ITCd weights. Each plot shows the temporal evolution of a single cell over four consecutive epochs, with the y-axis representing strength of activation. In the first and third epochs, the CS signals, shown in **(A)**, co-occur with *R*^+^, the US. In the second and fourth epochs, one of the CS signals is presented without *R*^+^. The red dotted lines indicate presence of *R*^+^. The green lines indicate extinction epochs in which *R*^+^ = 0. (Parameters: strength of inhibition *f*_*I*_ = 3.0; input from IL to ITCd *E*_ILd_ = 0; input from IL to ITCv *E*_ILv_ = 2).

However, the apparent redundancy of learning at the weights onto ITCv is unmasked in other circumstances. To demonstrate this, we modify the fear extinction paradigm. In the next set of simulations, only one of the two overlapping CS signals is extinguished. A subset of cells in CTX, LA and BA are therefore activated during both CS presentations, due to the aforementioned overlap. For example, let CS1 be an auditory signal containing frequencies from 500 to 1500 Hz, and let CS2 be a signal containing frequencies from 1000 to 2000 Hz. The two CSs overlap in the range from 1000 to 1500 Hz. During the extinction epochs, the cells in LA and BA that are activated during both CS presentations are subject to conflicting affective outcomes—they co-occur with both the US (*R*^+^) and its unexpected absense (*R*^−^). The cells in the amygdala circuit representing the range of overlap convey ambiguous information to the CeM cell. We therefore describe the situation as one of “confusing outcomes.” Thus, during extinction epochs the weights from LA onto ITCd and from BA onto ITCv that correspond to these overlapping cells in LA and BA take on fluctuating, intermediate values. These weights are alternately increased and decreased by the US and its absense, respectively. Because of these intermediate weight values, during extinction epochs the activities of ITCd and ITCv do not clearly distinguish the extinguished CS from the non-extinguished CS. This can be seen in the simulation results. In Figures 9-II-A and III-A, the ITCd activity takes non-zero values during presentation of both the extinguished CS and the non-extinguished CS. In Figures 9-I-B and III-B, the ITCv activity takes non-zero values during presentation of both the extinguished CS and the non-extinguished CS.

In Figure 9-I there is no learning in the LA-ITCd weights. Once again we see weaker magnitude CeM responses. In Figure 9-II there is no learning in the BA-ITCv weights, but here we see that fear responding to the second CS signal has not been extinguished at all. In Figure 9-III learning occurs in both sets of weights, and we see correct extinction learning, along with higher magnitude CeM activity. The activities of ITCv and ITCd—which are each ambiguous on their own—act synergistically to improve the performance of the system.

These results suggest the possibility that ITCd and ITCv—and by extension, their cortical inputs—play roles in contrast-enhancement, or in modifying the system's effective signal-to-noise ratio. In other words, the fear enhancing or suppressing roles of ITCd and ITCv might not function purely as on-off switches, but may also supplement the signal processing and filtering steps that occur at prior stages in the circuit.

#### 5.2.4. Attention and generalization

**Figure 10:**
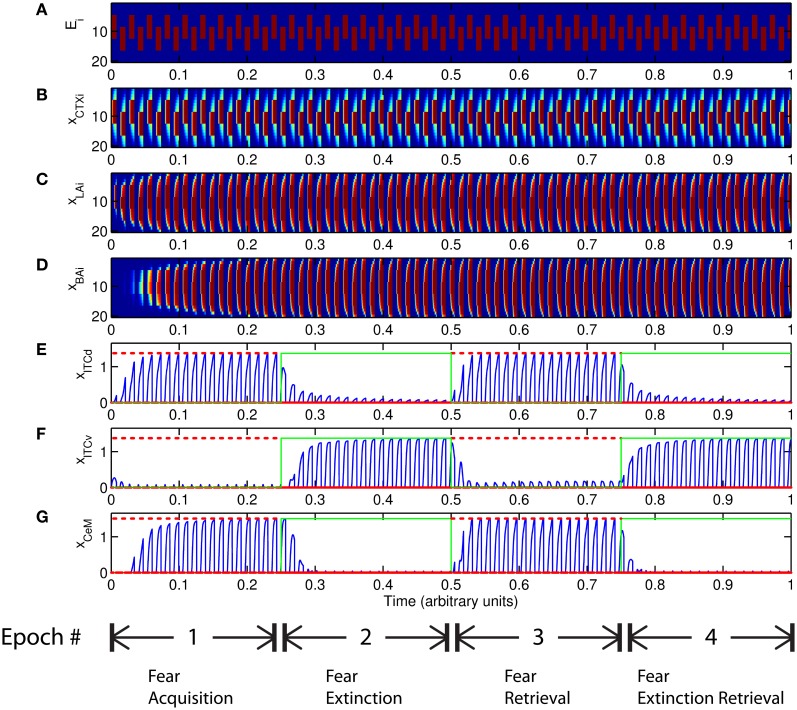
**Time evolution of model cell activities during “generalization” mode.** Both CS signals are extinguished. Plots show the time evolution of model cell activities during four consecutive epochs. Each plot **(A–D)** shows the temporal evolution of an array of 20 cells, with the y-axis representing the cell index, and the color representing strength of activation (Blue is low, red is high). Each plot **(E–G)** shows the temporal evolution of a single cell, with the y-axis representing strength of activation of that cell. In the first and third epochs, the CS signals co-occur with *R*^+^, the US. In the second and fourth epochs, the CS signals are presented without *R*^+^. (Parameters: strength of inhibition *f*_*I*_ = 0.3; input from IL to ITCd *E*_ILd_ = 0; input from IL to ITCv *E*_ILv_ = 2).

**Figure 11:**
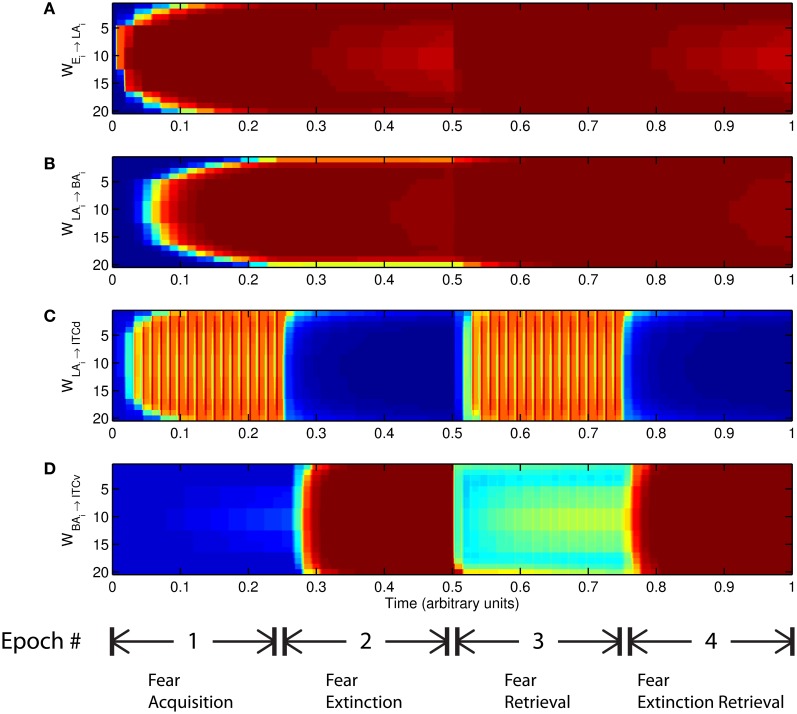
**Time evolution of model synaptic weights during “generalization” mode.** Both CS signals are extinguished, but weights corresponding to stimuli that were never presented are also increased. Each plot **(A–D)** shows the temporal evolution of an array of 20 connection weights, with the y-axis representing the index of the corresponding cell, and the color representing connection strength (Blue is low, red is high). In the first and third epochs, the CS signals co-occur with *R*^+^, the US. In the second and fourth epochs, one of the CS signals is presented without *R*^+^. (Parameters: strength of inhibition *f*_*I*_ = 0.3; input from IL to ITCd *E*_ILd_ = 0; input from IL to ITCv *E*_ILv_ = 2).

**Figure 12:**
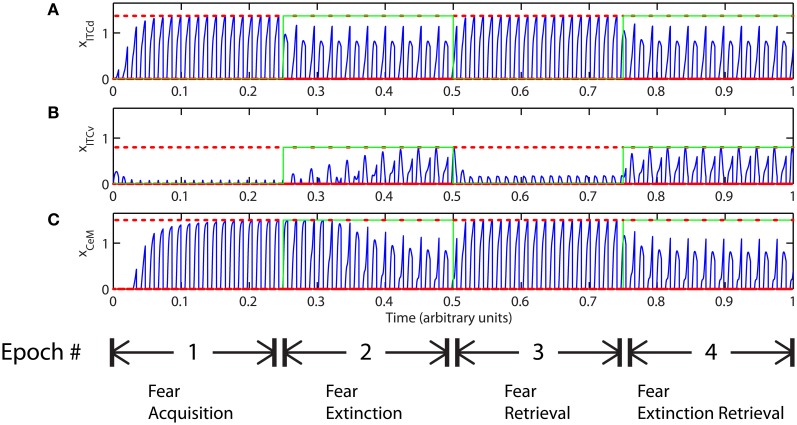
**Time evolution of model cell activities during “phobic” mode.** Only one of the two overlapping CS signals is extinguished. Each plot **(A–C)** shows the temporal evolution of a single cell over four consecutive epochs, with the y-axis representing strength of activation. In the first and third epochs, the CS signals, shown in **(A)**, co-occur with *R*^+^, the US. In the second and fourth epochs, one of the CS signals is presented without *R*^+^. The red dotted lines indicate presence of *R*^+^. The green lines indicate extinction epochs in which *R*^+^ = 0. (Parameters: strength of inhibition *f*_*I*_ = 0.3; input from IL to ITCd *E*_ILd_ = 0; input from IL to ITCv *E*_ILv_ = 2).

As described above, in certain configurations the CTX network may be unable to form a sharp representation of the CS. There may be contexts in which top–down attentional resources are overtaxed or spread too thinly. We model this low attention as weakened inhibition in the competitive-cooperative CTX network, leading to a spreading of activity, which in turn leads to spurious associations of the US with CSs that were not presented. If the outcomes are not “confusing,” learning can appear normal (Figures 10, 11). But if the outcomes are “confusing,” the system cannot extinguish learning, even if ILd is used to drive ITCd and put the network in “cautious” mode (Figure 12). Low attention prevents the system from discriminating between threatening and non-threatening stimuli, because the two have been categorized as the same via a process analogous to generalization.

## 6. Discussion

### 6.1. Implications of the simplified model

The simplified circuit model demonstrates some of the key emotional processes the rodent amygdala subserves—fear learning, extinction, and extinction retrieval. The model simulations demonstrate that if learning in the intercalated masses (ITCd and ITCv) is faster than in the BL complex (LA and BA), the system can rapidly switch between fear responding and extinction without discarding the CS-US associations in LA and BA. Cortical modulation from IL can be used to bias the system toward either a “rapid switch” mode or a “cautious” mode. Also, learning in ITCd and ITCv sometimes seems redundant, but in situations involving conflicting outcomes, these two regions may cooperate to disambiguate the incoming signal, prior to the final output stage of the amygdala. This synergy between ITCd and ITCv enhances the performance of the system in “confusing” situations, serving effectively as a form of information-processing. Cognitive control over the intercalated cells (ITCs in rodents, IM in primates) via IL/pOFC projections may serve as a mechanism to flexibly modify behavioral strategies in response to environmental contingencies. For instance, environments containing a mixture of appetitive and aversive stimuli may necessitate the “rapid switch” mode, so that the organism is brave enough to discover useful resources without being so foolhardy as to ignore threats. Extremely dangerous environments, on the other hand, may require the “cautious” mode, and an attenuation of exploratory behavior. Environments that are “confusing,” containing stimuli that are hard to distinguish, or whose affective consequences change over time, may require cortical or hippocampal (e.g., Frankland et al., 1998) enhancement of the information-processing abilities of the intercalated cells. The model demonstrates in simplified form how the amygdala and IL/pOFC can flexibly readjust fear responses as contingencies change—such roles have also been inferred from human fMRI research (Schiller et al., 2008).

The model also allows us to demonstrate that weakened top–down attention can prevent the system from discriminating between threatening and non-threatening stimuli. This occurs via a process of over-generalization, in which two stimuli cannot be separated on the basis of their affective consequences. Though we posit that this over-generalization occurs as a result of cortical mechanisms, the basic process may also be applied to model the pathological over-generalizations that have been attributed to hippocampal dysfunction (reviewed in (Kheirbek et al., 2012). Pathological over-generalization may also have a basis within the amygdalar circuit (Mahan and Ressler, 2012). Patients diagnosed with PTSD display over-generalization (e.g., Lissek and Grillon, 2010), and this could result from the kind of attentional dysfunction employed in the model. But it is premature to extrapolate from our simplified model to complex human psychological phenomena. Nevertheless, the modeling results can be used to guide hypotheses to be explored further in experimental animals and humans. For instance, medical interventions that enhance attention may allow patients with PTSD to better discriminate between threatening and non-threatening stimuli.

The modeling results show that learning in a simplified subset of the possible amygdalar network connections is sufficient to exhibit flexible emotional learning. More complex tasks will be necessary to resolve the roles of seemingly redundant connections or representations in the network. Future anatomical and physiological studies will allow us to make more specific claims about the nature and location of synaptic changes, and also about the neurochemical signals that are necessary for these changes to occur.

More generally, the approach here shows that simple simulations of a neural circuit constructed from the bottom up not only agree with experimental findings, but also suggest and predict novel roles for network elements that go beyond straightforward extrapolations from experiment. For instance, the model simulations point to possible information-processing roles for the ITCs. The model suggests that these cells are not simply on-off switches for the fear response, but can act synergistically to enhance the model's ability to discriminate between stimuli in situations that are confusing. Further, since the ITCs receive projections from prefrontal cortex, they may be part of a circuit for top–down effects on emotional expression and suppression. These results are consistent with an earlier biophysical modeling study by Li et al. (2011), which shows that IL can overcome inter-ITC inhibition to control CeM output. That study is the only other computational model that explicitly incorporates the ITCs. As we have done here, Li et al. (2011) also omitted the effects of CeL due to paucity of data.

Another comparable computational model is that of Krasne et al. (2011). Their amygdalar model is also rate-coded and incorporates learning, but has a complementary focus—one of their central modeling targets is an extensive exploration of hippocampus-dependent contextual fear conditioning. Our model does not incorporate hippocampal connections, but uniquely highlights the possible effects of IL projections to ITCs. The model of Krasne et al. (2011) posits that extinction takes place via interneurons in BLA, whereas we propose that extinction occurs due to learning at the ITCs. Their computational approach also differs: their implementation can be described as algorithmic and algebraic, whereas ours is based on dynamical systems.

Thus, we show that a rate-coded model can corroborate and extend insights gained from more fine-grained biophysical spiking models such as that of Li et al. (2011), and can also complement other higher-level approaches such as that of Krasne et al. (2011). Simplified models such as ours also have the benefit of greater computational tractability than biophysical models, allowing for rapid investigation of qualitative circuit-level phenomena. Further, our model is the only one we are aware of to incorporate synaptic learning at the ITCs. The information-processing role predicted by our model may be linked with the integrative roles for ITCs proposed by Palomares-Castillo et al. (2012).

Our model shows how the amygdalar local circuit depicted in Figure 3 can assign emotional significance to stimuli and use these categorized stimuli to drive emotional behavior such as the freezing response. The model is based on circuits described in rodents, in which behavioral and physiological data are available. As discussed above, in rhesus monkeys the principal pathway from the cortex to IM (the primate equivalent to rodent ITCs) originates in pOFC (Ghashghaei and Barbas, 2002). In rhesus monkeys IM neurons are not segregated into dorsal and ventral clusters, but belong to at least three neurochemical classes of inhibitory neurons which are intermingled within IM (Zikopoulos and Barbas, 2011). The distinct classes of inhibitory neurons may have critical, and perhaps specific, roles in emotional arousal and return to autonomic homeostasis.

### 6.2. Future directions: extending the model

Ongoing work will incorporate more of the local amygdalar circuit connections, and embed the amygdala more fully into the cognitive-emotional continuum we described earlier. We aim to progressively expand the computational model, so that it can tie together more of the experimental data, display more diverse, flexible behaviors, and suggest neural accounts of psychiatric disorders that can inform translational research.

In order to form a more nuanced picture of the relationship between emotion and attention, it is necessary to address the fact that attention-related neural mechanisms can both affect the amygdalar circuit and be affected by amygdalar outputs. In keeping with this goal we hope to include in future iterations of the model the recently discovered projections from amygdala to the thalamic reticular nucleus (TRN) (Zikopoulos and Barbas, 2012). Thalamic processing for the suppression of irrelevant stimuli is crucial for selective attention, and may be accomplished early in neural processing through the inhibitory TRN. The TRN lies between the thalamus and cortex and plays a key role in processes that direct attention to relevant/significant stimuli (Figure 1). The TRN receives projections from all cerebral cortices and their associated thalamic nuclei, but sends inhibitory output only to the thalamus, effectively gating thalamo-cortical communication (Crick, 1984; Montero, 1997; Weese et al., 1999; Pinault, 2004; McAlonan et al., 2008; Petrof and Brown, 2010). Projections from sensory and motor cortices and their thalamic nuclei map topographically on TRN (reviewed in (Guillery and Harting, 2003; Pinault, 2004; Zikopoulos and Barbas, 2007). In primates, prefrontal cortices innervate the anterior sector of TRN (Zikopoulos and Barbas, 2006). However, lateral prefrontal cortex (areas 46 and 9) and pOFC, which are major sensory-recipient prefrontal regions, and their associated thalamic nucleus, the mediodorsal (MD), have widespread projections that extend beyond the frontal sector of TRN to sites innervated by sensory and motor cortices (Zikopoulos and Barbas, 2006). Through this unique type of projection, lateral prefrontal and posterior orbitofrontal cortices may control the passage of signals through the thalamus to shift attention to relevant stimuli and suppress distracters (Barbas and Zikopoulos, 2007). The amygdala may be in a position to modulate this attentional mechanism via a novel and robust excitatory pathway from the basal amygdala that also innervates widely the inhibitory TRN in rhesus monkeys (Zikopoulos and Barbas, 2012). This pathway innervates the entire antero–posterior axis of TRN, and converges at sites that receive widespread projections from MD, pOFC and lateral prefrontal cortices. An additional distinguishing feature of this amygdalar pathway is the presence of large and efficient synapses that target TRN neurons proximally. This unique and widespread pattern of connectivity suggests that this system is suited for an overseeing role in events that require heightened attention to stimuli that are essential for survival, or simply for rapid attention to salient stimuli to make a judgment for a course of action.

Attentional and emotional processes are also linked via the widely projecting neurotransmitter systems (relevant human studies are reviewed in (Davis and Whalen, 2001). For instance, the cholinergic projection system in the basal forebrain, which includes the nucleus basalis of Meynert (NBM) and the substantia innominata (SI), may play an important role in the interactions between the amygdala and the prefrontal cortex. The amygdalar central nucleus (Ce) and the IM (ITCs in rodents) project to the basal forebrain (Paré and Smith, 1994; Bourgeais et al., 2001). These cholinergic pathways from the basal forebrain may have widespread effects on the entire cortex, and may affect general vigilance through tonic signals, or enhance attention through phasic activity (Davis and Whalen, 2001; Sarter and Parikh, 2005; Parikh and Sarter, 2008). Among prefrontal cortices the ACC and the pOFC receive the strongest cholinergic projections from the basal forebrain (Mesulam et al., 1992; Ghashghaei and Barbas, 2001).

It has long been established that both emotional salience and direct stimulation in the amygdala promote memory formation (McGaugh, 2004; Chau and Galvez, 2012), a process which may involve the substantial projections from midbrain dopaminergic areas to amygdalar nuclei (Björklund and Dunnett, 2007; Cho and Fudge, 2010). Dopaminergic signals can serve as teaching signals that affect synaptic plasticity and memory, often in conjunction with other neurotransmitters (Nader and LeDoux, 1999; LaLumiere et al., 2004). More recent studies have established that DA is necessary for normal learning of cued fear responses, and that an absence of normal DA signaling during fear conditioning instead leads to the development of generalized anxiety (Zweifel et al., 2011).

In summary, incorporating the TRN and the neurotransmitter systems may allow us to expand our simplified attentional mechanism, and also investigate teaching signals and synaptic plasticity, both within the amygdala and in regions affected directly and indirectly by amygdalar output. These pathways to and from the amygdala may be well suited to serve as the basis of a more general phenomenon of emotional “perception” (Vuilleumier et al., 2004; Hadj-Bouziane et al., 2008; Lim et al., 2009; reviewed in, Barrett and Bar, 2009).

## 7. Conclusion

We have argued that rather than being opposing forces, cognition, and emotion can be seen as points on a continuum or gradient of flexible processes required for adaptive categorization of, and response to, changes in the external and internal environment of an organism. While this conceptualization may not capture all the psychological nuances of the terms, it highlights the experimentally tractable facets of “cognition” and “emotion.”

The functional continuum is based on the robust connections between areas associated with cognition and those associated with emotion. The amygdala and the pOFC receive coarse-grained information from a variety of brain regions, and are both in a position to integrate internal and external environmental signals into broad emotion-related representations of stimuli and overall context. The pOFC sends “feedback” projections to lateral prefrontal cortices, which are associated with cognition, and receive fine-grained sensory information. Compared with pOFC, lateral prefrontal cortices may thus form more precise representations of the environment. Such representations can then be sent via “feedforward” projections to areas associated with emotions and goal-directed behavior (pOFC), from which they can influence internal states and behavior through specialized projections to the amygdala.

Our simplified computational model illustrates one way that the amygdala can carry out emotional categorization and response-generation. The model also shows how the prefrontal cortex, acting via the intercalated cell groups, can modulate learned fear responding, and facilitate flexible switching between fear expression and suppression without loss of prior learning. The prefrontal cortical projections can put the system in a “cautious” mode in which fear cannot be suppressed, or in a “rapid-switch” mode in which extinction is sped up. “Reducing” the level of attention in the model provides a mechanism by which the system can generalize the consequences of a stimulus, or enter into a “phobic” mode that is resistant to extinction. In future studies attentional modulations may be incorporated into the model by adding the known projections of amygdala to TRN and the neuromodulatory systems. Thus, emotional categorization of stimuli that arrive at the amygdalar circuit not only drives responses, but can also lead to widespread changes in high-level cortical processing.

In conclusion, the model demonstrates how a computational approach can suggest non-trivial functional roles of network components. Among these, the model simulations reveal an information-processing role for intercalated neurons in learning emotional associations and flexibly altering expectations when stimuli no longer signal a threat (or lack of reward) in the environment. The computational model based on key nodes in the amygdalar circuit also provides a plausible mechanism for the generalization of stimuli, which may underlie the pattern of activation in the amygdalar-prefrontal circuit in a variety of anxiety disorders including phobias and PTSD.

### Conflict of interest statement

The authors declare that the research was conducted in the absence of any commercial or financial relationships that could be construed as a potential conflict of interest.

## Abbreviations

AA: anterior amygdaloid area
ACC: anterior cingulate cortex
Amy: amygdala
aOFC: anterior orbitofrontal cortex (primates)
BA: basal nucleus of amygdala
BLA: basolateral amygdala
BMA: basomedial amygdala
Ce: central nucleus of amygdala
CeL: lateral subdivision of Ce
CeM: medial subdivision of Ce
CS: conditioned stimulus
CTX: cortex (model)
IL: infralimbic cortex (rodents)
ILd: projection from IL to ITCd (rodents)
ILv: projection from IL to ITCv (rodents)
IM: intercalated masses (primates)
ITC: intercalated cells (rodents)
ITCd: dorsal intercalated cell group (rodents)
ITCv: ventral intercalated cell group (rodents)
LA: lateral nucleus of amygdala
MD: mediodorsal nucleus of thalamus
Me: medial nucleus of amygdala
NBM: nucleus basalis of Meynert
PL: prelimbic cortex (rodents)
pOFC: posterior orbitofrontal cortex (primates)
SI: substantia innominata
SNc: substantia nigra pars compacta
Thal: thalamus
TRN: thalamic reticular nucleus
US: unconditioned stimulus
VCo: ventral cortical nucleus of amygdala
VTA: ventral tegmental area.

## Appendix

### Methods

Our modeling approach can be described as “phenomenological”—the equations governing neural activity and synaptic weight change are not intended to accurately reflect fine-grained biophysical detail or fit quantitative experimental data. Instead, our simplified model provides qualitative results that agree with several experimental studies, and also allow us to suggest possible functional roles for components of the network. As described in the Introduction, this approach can address several questions related to how the components of the amygdalar local circuit work together as a substrate for flexible fear-related learning and responding.

We implement the model as a rate-coded system to investigate those properties of the amygdalar network that depend on connectivity, rather than fine-grained biophysical details. We assume as a first approximation that all the model neurons obey the same set of differential equations. We assign an activity *x*_*s*_ to each neuron *s*:

(A1) $$\tau_{x_{s}}\frac{dx_{s}}{dt}=-A_{s}x_{s}+(B_{s}-x_{s})E_{s}-(x_{s}+C_{s})I_{s}$$

where subscript *s* is an index for the particular neuron in the network, and *E*_*s*_ and *I*_*s*_ correspond to the excitatory and inhibitory inputs, respectively. The term *A* specifies the passive decay rate, *B* is the maximum activity (*B* > 0), and −C is the minimum activity (*C* ≥ 0). The time constant of integration is τ_*x*_*s*__.

Synaptic weight changes occur via the following learning rule:

(A2) $$\tau_{c}\frac{dW_{c}}{dt}=\left\{-A_{c}W_{c}+R(W_{m}-W_{c})\right\}{\left[x_{\text{pre}}\right]}^{+}{\left[x_{\text{post}}\right]}^{+}$$

where *W*_*c*_ is a connection weight between two neurons, *R* is a reinforcing signal, *x*_pre_ is the presynaptic neural activity, and *x*_post_ is the postsynaptic neural activity. The constant *W*_*m*_ is the maximum attainable weight. The square brackets indicate positive rectification (if *q* ≥ 0, [*q*]^+^ = *q*, else [*q*]^+^ = 0). The weight change is presynaptically and postsynaptically gated. Weight *W*_*c*_ increases if *x*_pre_ and *x*_post_ take non-zero values and the teaching signal *R* is simultaneously greater than zero. Weight *W*_*c*_ decreases if *x*_pre_ and *x*_post_ take non-zero values, but there is no accompanying teaching signal, i.e., *R* = 0. Parameter *A*_*c*_ represents a rate of gated decay or “active forgetting.” Subscript *c* is an index that specifies the connection (see Table A1 for a complete list). For example, in Table A1, *W*_CTX__i → LA_i__ corresponds to the connection weight from the *i*th cortical cell to the corresponding LA neuron.

**Table A1:** **Excitatory and inhibitory inputs to each network component**.

**Cell index**	**Excitation term *E*_*s*_**	**Inhibition term *I*_*s*_**
CTX_*i*_	$E_{i}+\sum_{k=1}^{N}W_{{\text{CTX}}_{k}\to {\text{CTX}}_{i}}^{E}E_{k}$	$\sum_{k=1}^{N}W_{{\text{CTX}}_{k}\to {\text{CTX}}_{i}}^{I}E_{k}$
LA_*i*_	*f*_LA_*W*_CTX_*i*_ → LA_*i*__ [CTX_*i*_]^+^	0
BA_*i*_	*W*_LA_*i*_ → BA_*i*__ [*x*_LA__*i*_]^+^	0
ITCd	$\sum_{i=1}^{N}W_{{\text{LA}}_{i}\to \text{ITCd}}{\left[x_{{\text{LA}}_{i}}\right]}^{+}+E_{\text{ILd}}$	0
ITCv	$\sum_{i=1}^{N}W_{{\text{BA}}_{i}\to \text{ITCv}}{\left[x_{{\text{BA}}_{i}}\right]}^{+}+E_{\text{ILv}}$	*W*_ITCd → ITCv_ [*x*_ITCd_]^+^
CeM	$\sum_{i=1}^{N}{\left[x_{{\text{BA}}_{i}}\right]}^{+}$	*W*_ITCd → CeM_ [*x*_ITCv_]^+^

Let there be *N* cells each in CTX, LA, and BA. Each CTX neuron receives one input *E*_*i*_ (where *i* is an index that runs from 1 to *N*). Each *E*_*i*_ is a neutral stimulus that can, after learning, come to modulate an emotional response. Each *E*_*i*_ can represent a point in a feature space, such as a particular auditory frequency. Each CTX cell sends excitatory projections to nearby CTX cells, as well as inhibitory projections, which can be interpreted as taking place via intermediary inhibitory interneurons, as depicted in the top inset of Figure 3. Parameters are chosen so that the CTX cells work together as a distance-dependent on-center off-surround network. Excitatory connections between CTX cells are determined by a Gaussian, such that

(A3) $$W_{{\text{CTX}}_{k}\to {\text{CTX}}_{i}}^{E}=e^{-{\left[(k-i)/\sigma_{E}\right]}^{2}}$$

Similarly, inhibitory connections between CTX cells are given by

(A4) $$W_{{\text{CTX}}_{k}\to {\text{CTX}}_{i}}^{I}=f_{I}e^{-{\left[(k-i)/\sigma_{I}\right]}^{2}}$$

where *f*_*I*_ governs the strength of inhibition.

Each CTX cell projects to a corresponding LA cell, resulting in a one-to-one topographic mapping. Similarly, each LA output in turn serves as an input to a corresponding BA neuron. All subsequent processing stages involve a convergence or fan-in of activity from LA and BA. There is a single activity corresponding to each of the following regions: ITCd, ITCv, and CeM. The excitatory input to ITCd consists of a weighted sum of inputs from LA. The excitatory input to ITCv consists of a weighted sum of inputs from BA. ITCd inhibits ITCv. The excitatory input to CeM consists of a sum of inputs from BA. The inputs to CeM are not weighted, and are not subject to synaptic change.

The activities of the artificial neurons in Figure 3 are each governed by equation A1, with the excitation term *E*_*s*_ and inhibition term *I*_*s*_ specified in Table A1. Weight change occurs via equation A2 in the following sets of weights: *W*_CTX__*i*_, *W*_LA__*i* → BA_*i*__, *W*_LA__*i* → ITCd_, and *W*_BA__*i* → ITCv_. The teaching signal *R* for the first three of these sets is given by *R*^+^, which takes a value of 1 when the US is present, and is zero otherwise. The teaching signal *R*^−^ for *W*_BA__*i* → ITCv_ is assumed to carry complementary information to *R*^+^, and is given by:

(A5) $$R^{-}=(1-R^{+})\text{sgn }\left\{\sum_{i=1}^{N}{\left[x_{{\text{BA}}_{i}}\right]}^{+}\right\}$$

This term can also be described as a binary expectation-violation signal. If the sign of the sum of BA activities is interpreted as a long-term expectation of the US co-occurring with the CS, then *R*^−^ takes non-zero values when there is a discrepancy between the outcome and the expectation encoded by the BA activities, i.e., when [x_BA__*i*_]^+^ take non-zero values, but there is no US signal, so *R*^+^ = 0.

### Simulation details

In all the simulations shown here, parameters take the values specified in Table A2. The equations are integrated using the simple Euler method, with a step size of 0.00001. The simulations are run for 40,000 time steps, so each epoch lasts for 10,000 time steps. The CSs (CS1 and CS2) and US are rectangular pulses. The onset of US occurs at the half-way point of the CS. The CS and US are reset to zero simultaneously. Activities *x*_*s*_ begin at zero, and are reset whenever the CS signal returns to zero. The values of the strengths of inhibition *f*_*I*_, excitatory input *E*_ILd_ from IL to ITCd and excitatory input *E*_ILv_ from IL to ITCv vary across different simulations, as specified in the corresponding figure captions.

**Table A2:** **Parameter values common to all simulations**.

**Parameter**	**Value**
*N*	20
*E*_*i*_	10 (peak value)
τ_*x*_	0.001
*A*_CTX*i*_	0.1
*B*_CTX*i*_	2
*C*_CTX*i*_	2
*A*_LA_*i*_, BA_*i*_, ITCd, ITCv, CeM_	10
*B*_LA_*i*_, BA_*i*_, ITCd, ITCv, CeM_	2
*C*_LA_*i*_, BA_*i*_, ITCd, ITCv, CeM_	0
*f*_LA_	10
τ_CTX_*i*_ → LA_*i*__	0.0016
*A*_CTX_*i*_ → LA_*i*__	0.001
τ_LA_*i*_ → BA_*i*__	0.0066
*A*_LA_*i*_ → BA_*i*__	0.001
τ_LA_*i*_ → ITCd_	0.133
*A*_LA_*i*_ → ITCd_	50
τ_BA_*i*_ → ITCv_	0.133
*A*_BA_*i*_ → ITCv_	50
*W*_*m*_	20
*W*_ITCd → ITCv_	10
*W*_ITCv → CeM_	25
*W*_*s*_(*t* = 0)	0.05

Terms with “(t = 0)” specify initial values.
